# Porous liquids – the future is looking emptier

**DOI:** 10.1039/d2sc00087c

**Published:** 2022-04-25

**Authors:** Benjamin D. Egleston, Austin Mroz, Kim E. Jelfs, Rebecca L. Greenaway

**Affiliations:** Department of Chemistry, Molecular Sciences Research Hub, Imperial College London White City Campus, 82 Wood Lane London W12 0BZ UK r.greenaway@imperial.ac.uk k.jelfs@imperial.ac.uk

## Abstract

The development of microporosity in the liquid state is leading to an inherent change in the way we approach applications of functional porosity, potentially allowing access to new processes by exploiting the fluidity of these new materials. By engineering permanent porosity into a liquid, over the transient intermolecular porosity in all liquids, it is possible to design and form a porous liquid. Since the concept was proposed in 2007, and the first examples realised in 2015, the field has seen rapid advances among the types and numbers of porous liquids developed, our understanding of the structure and properties, as well as improvements in gas uptake and molecular separations. However, despite these recent advances, the field is still young, and with only a few applications reported to date, the potential that porous liquids have to transform the field of microporous materials remains largely untapped. In this review, we will explore the theory and conception of porous liquids and cover major advances in the area, key experimental characterisation techniques and computational approaches that have been employed to understand these systems, and summarise the investigated applications of porous liquids that have been presented to date. We also outline an emerging discovery workflow with recommendations for the characterisation required at each stage to both confirm permanent porosity and fully understand the physical properties of the porous liquid.

## Abstract 2

The realisation of permanent microporosity in liquids transforms the way functional porosity may be implemented. Considering recent advances, we explore the developing theory of porous liquids and delve into the discovery process and applications.

## Graphical abstract

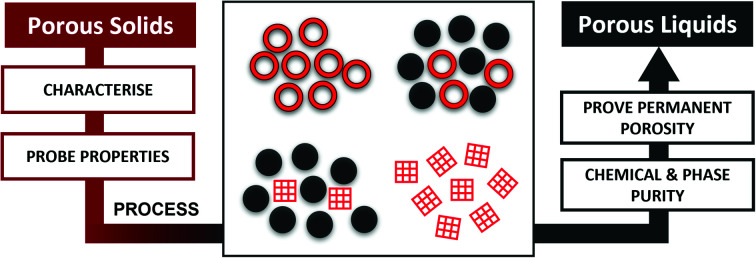

## 1. Introduction

**Fig. 1:**
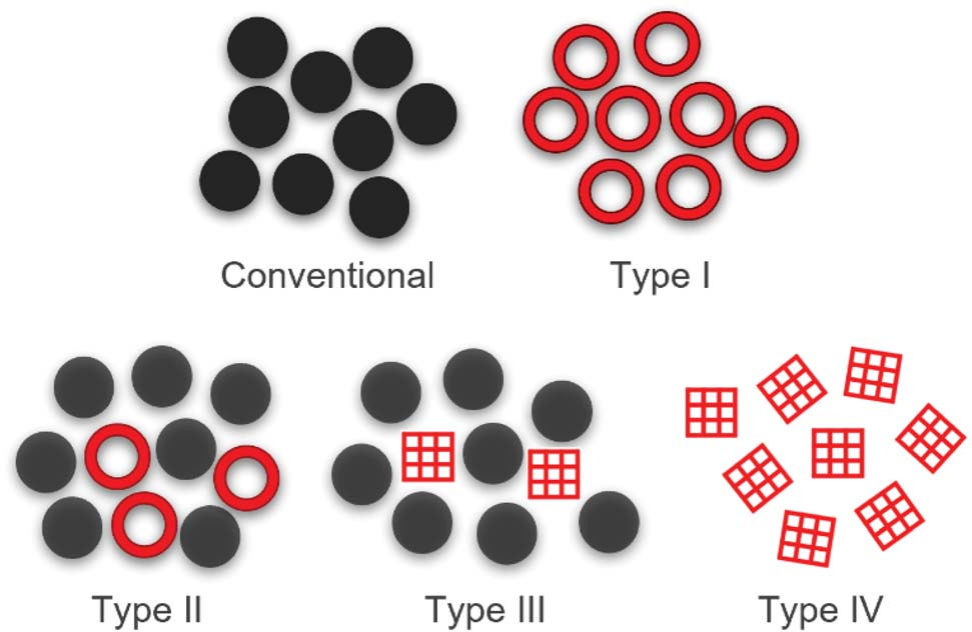
Representation of conventional liquids, which do not contain permanent intrinsic porosity, compared to the four proposed types of porous liquids: type I – neat molecular species containing permanent pores which are liquid at room temperature or low-melting; type II – solutions of microporous materials in cavity-excluded solvents; type III – dispersions of microporous solids in cavity-excluded liquids; and type IV – neat meltable microporous extended frameworks.

Microporous materials have widespread applications in gas capture,^1,2^ molecular separations,^3,4^ catalysis,^5,6^ and energy storage.^7–9^ Traditionally, these materials have been solid, but recently, permanent porosity has been translated into the liquid state. All conventional liquids exhibit transient porosity to some extent due to a liquid's disordered and dynamic structure, though these pores are temporary and have poorly defined shape and size.^10^ Porous liquids combine the mobility of liquids with the properties of a microporous solid, and fundamentally differ from conventional liquids in that they contain permanent, accessible cavities. Since they were first conceptualised by James and co-workers,^11^ a range of different strategies have been employed to incorporate permanent, shape-persistent cavities and to engineer ‘intrinsic’ porosity into liquids, rather than the transient ‘extrinsic’ porosity seen in all liquids.^12–16^ While not all systems fit neatly into these categories, four types of porous liquids have now been proposed (Fig. 1): type I – neat molecular porous liquids; type II – solutions of molecular porous species in pore-excluded solvents; type III – dispersions of microporous solids in pore-excluded liquids; and type IV – neat meltable microporous extended frameworks.^11,16^

In porous liquids, discussing intrinsic porosity slightly differs from the use in describing porous molecular solids. Intrinsic porosity in a solid arises when the structures of discrete, individual molecules possess an internal void or internal free volume resulting in voids in the bulk material, whereas extrinsic porosity arises when molecules that do not have this intrinsic void or free volume instead form a porous network from spaces between molecules due to the packing in the bulk material. In contrast, all porous liquids must contain some permanent intrinsic porosity originating from a pore carrier, and pores can also arise in a porous liquid due to formation of transient voids in the material external to the pore carrier. For example: in type I porous liquids there may be transient porosity between the molecular species that contain the intrinsic porosity; in type II porous liquids, the size-excluded solvent may contain transient porosity with the pore carrier providing the permanent intrinsic porosity; and in type III porous liquids, likewise the size-excluded solvent may contain transient porosity and the solid pore carrier provides the intrinsic porosity in the porous liquid. However, according to the nomenclature of molecular porous solids, this can include both intrinsic and extrinsic porosity when intrinsically porous molecules pack in the solid component giving rise to extrinsic pores. In type IV systems, the porous frameworks provide the intrinsic porosity in the porous liquid.

The porosity in a porous liquid should also be permanent – this means that any solvent used to form the porous liquid should be size-excluded from the cavities and should not be simply weakly bound in the cavity and displaced by guest molecules due to favorable host–guest interactions. In addition, the shape-persistent cavities within a porous liquid should be accessible to guest molecules, which for example, rules out solutions of fullerenes (molecular compounds which contain voids but do not include any windows to access the cavity) as porous liquids, although they conceptually meet the initial definition in that permanent pores are present. One could also argue that the addition of a guest, in particular gases, should be reversible in order to quantify the nature of the porosity, whether that is by using a temperature- or pressure-swing as is frequently used in porous solids, or by other controllable release mechanisms. There should also be a demonstrable increase in porosity over the neat or a directly comparable non-porous liquid – this means that reasonable cavity concentrations need to be achieved, for example, this is particularly relevant in type II porous liquids. Finally, the porous liquids should ideally be in the liquid state at or near room temperature, or at least at some operating temperature of a process in which it can be applied, although the gas uptake will significantly reduce at higher temperatures. Overall, this leads us to propose that the following general criteria should ideally be met for a liquid or porous material to be classed as a ‘porous liquid’, or a permanently microporous liquid, and could act as a checklist for future researchers:

• Contains permanent intrinsic porosity from a pore carrier

• Intrinsic porosity must be accessible to guests in order to demonstrate the nature of the porosity

• Has a sufficiently high pore volume that it exhibits a demonstrable guest uptake

• Liquid at or near room temperature, or at a specified operating temperature

Like their solid counterparts, porous liquids are capable of both gas uptake and selectivity, and many demonstrate increased gas solubility compared to conventional liquids. However, porous liquids also offer unique properties and applications, such as the possibility of pumping in continuous systems, and facilitating guest loading and unloading steps. The current state-of-the-art for industrial gas capture and separation, such as in carbon capture and sequestration (CCS), relies on liquid chemical sorbents^17^ – there are many issues with retrofitting industrial systems for the use of solid microporous materials,^18,19^ but this could be remedied through use of porous liquids. Whilst there is still a long way to go in designing competitive systems, porous liquids have the potential to be more efficient alternatives to conventional liquid sorbents, for example, to remove CO_2_ from natural gas (CH_4_) and syngas (CO/H_2_),^20^ by exceeding both uptake and selectivity while requiring less energy for regeneration. In addition to this potential application in carbon capture, porous liquids could also have applications in catalysis and as gas-loaded solvents, and in molecular separations of non-gaseous species.

This review highlights the key milestones in the development of porous liquids and summarises the applications investigated to date, including the drive to form porous liquids with increasing porosity that exceed the capacity of systems reported prior to them. Beyond this, we also discuss the most widely used and useful experimental and computational techniques that have been employed to characterise and understand the properties of the different types of porous liquids to date. In doing so, we propose an outline for best practice and a minimum characterisation requirement at each stage of the porous liquid discovery process. Among other recent reviews, this work aims to lay out the key concepts and approaches that are important in developing and understanding porous liquids, rather than expanding on the classifications.^16^ However, previous reviews have predominantly focused on outlining the early examples of materials in this class and how new porous liquids may be developed,^13–15^ have given an extensive and exclusive summarisation of the porous liquid literature,^21^ or have focused on the important physical properties of porous liquids that make them candidates for certain applications.^22^ In contrast, we hope this provides readers with a short and accessible review to understand the parallel concepts employed in the discovery of porous liquids as well as the breadth of potential applications.

## 2. Key milestones in the development of porous liquids

**Fig. 2:**
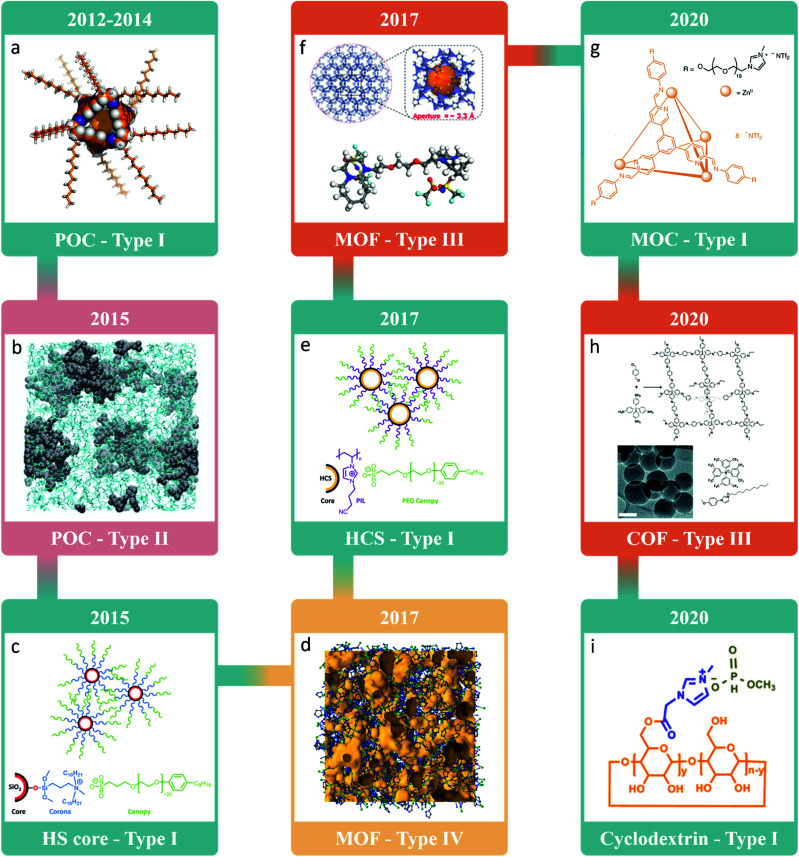
Timeline showing some of the key milestones and advances in the field from when the concept of porous liquids was first introduced, focusing on the first example of each type of porous liquid and/or use of different porous materials. (d) Reprinted by permission from Springer Nature, Nature Materials, ref. 57. Copyright 2017; (f) reprinted (adapted) with permission from ref. 40. Copyright 2018. American Chemical Society; (g) reprinted by permission from Springer Nature, Nature Chemistry, ref. 53. Copyright 2020; (h) reproduced from ref. 55 with permission from the Royal Society of Chemistry. Copyright 2020; (i) reprinted (adapted) with permission from ref. 56. Copyright 2020. American Chemical Society.

After the idea of porous liquids was introduced in 2007, it was many years before the first examples with both permanent porosity and demonstrable gas uptake were realised, highlighting the main challenge in developing porous liquids – maintaining the porosity of the solid whilst rendering it into a liquid state. Initial studies focused on the formation of neat porous liquids, presumably as type I systems potentially offer access to the highest pore concentration per unit volume. These first attempts were based on porous organic cages (POCs) – discrete molecules containing a permanent shape-persistent cavity accessible through windows. POCs typically decompose prior to melting, but peripheral alkyl groups can be introduced to suppress the melting temperature significantly (Fig. 2a).^23,24^ However, these liquids suffered from interpenetration, with the POC cavities spontaneously occupied by alkyl groups from neighboring cages, meaning porosity was lost – it was predicted that the *n*-pentyl decorated POC would maintain 30% of its porosity in the liquid state, but this was never experimentally validated. While this study did not yield a type I permanently porous liquid at room temperature, this was the first reported example of meltable shape-persistent POCs, which recently has led to the liquid phase being exploited in the formation of melt-quenched molecular organic cage glasses.^25^ In addition, while the formation of a melt-quenched glass with glass transition was not formally noted, it is also worthwhile to mention that one of the alkylated POCs from the initial type I porous liquid study was reported to also have a glassy appearance, namely the *n*-octyl decorated POC.^21^

It was not until 2015 that the first systems formally identified as porous liquids were reported, where both type I and type II porous liquids, based on hollow silica spheres and POCs respectively, were realised.^26,27^ Building on the initial attempts at forming porous liquids using POCs, two different strategies were employed to improve their solubility, as opposed to inducing melting, enabling high concentration type II porous liquids to be formed on dissolution in cavity-excluded solvents (Fig. 2b).^26^ The first approach involved decorating POCs with crown-ether functionality to increase their solubility in 15-crown-5 in a ‘like-for-like’ strategy, and the second involved forming mixtures of vertex-disordered mixtures of POCs to disrupt the solid-state packing and therefore increase solubility in the size-excluded solvent hexachloropropene, in a ‘scrambling’ strategy. Both of these systems exhibited increased methane uptake compared to their respective neat solvents,^26^ with an in-depth study of the latter system later expanding on the range of gases that could be absorbed, and providing a deeper understanding of the guest selectivity and diffusion in the porous liquid.^28^ Overall, the dissolution of POCs in size-excluded solvents has proven itself to be a promising method of generating porous liquids whose properties can be modified based on both the selected POC and solvent. For example, a family of ‘scrambled’ type II porous liquids, where the functionality on the POC vertices and the size-excluded solvent were varied, was reported in 2019 after development of a high-throughput workflow to rapidly accelerate their discovery.^29^ Of particular note, higher pore volumes were theoretically accessible based on the discovery of more soluble POC/solvent combinations, increasing from ∼0.65% pore volume for a 20% w/v porous liquid to 1.12% and 1.49% for 40% and 60% w/v porous liquids respectively – these calculations were based on the average cavity size of the POCs and the density of the porous liquid, but the extent of which each of the pore sizes was reduced based on the solvents occupying the window sites was not taken into account.^26,27^ Later in 2020, a POC with a narrower pore aperture was also dissolved in hexachloropropene, which enabled the gas absorption properties in type II porous liquids to be tuned for the first time.^30^

In comparison, the type I porous liquid also reported in 2015 adopted an alternative approach with fluidised hollow silica (HS) spheres – a surface engineering strategy was employed to incorporate suitable corona and canopy species affording a liquid at room temperature (Fig. 2c).^27^ While this system may not fit neatly into the category of type I porous liquids due to the larger particle sizes of the HS spheres containing mesopore cavities, at least based on the initial definition of them being neat microporous *molecular* liquids, the shell itself was microporous, preventing occupation of the core. In addition, the particles were homogenously combined with the corona–canopy species forming a neat liquid with pores that is distinct to colloidal suspensions which would fit more with a type III porous liquid. As with the type II porous liquids described above, this strategy was subsequently applied to other systems. For example, silica nanoparticles functionalised with different corona and canopy species were reported as additional examples of type I porous liquids;^31,32^ nanosized silicalite-1 zeolite particles were subjected to a surface sol–gel process to form particles decorated with organosilanes (OS) and poly(ethylene glycol) (PEG) tails;^33^ hollow silica nanorods were covalently functionalised with an OS canopy before electrostatic grafting of a polymer surfactant to afford a solvent-free mesoporous liquid;^34^ and metal–organic frameworks (MOFs) were surface-modified with OS and oligomeric species *via* covalent bonding to form a porous fluid at room temperature.^35,36^ In addition, similar approaches were later demonstrated with hollow carbon spheres (HCS) decorated with polymeric ionic liquids by exploiting the electrostatic interaction between the two species (Fig. 2e).^37,38^

In contrast to the other types of porous liquids where there are now a handful of examples of each known, there has been a large surge of interest in type III porous liquids, likely driven by the ‘mix-and-match’ strategy that can be employed in combining the wide range of known solid microporous materials with a whole host of different liquids to form dispersions. A certain level of design is still required for these systems however, with the requirement for the liquid to be excluded from the porous material and the formation of stable dispersions desired. Arguably, the first example of a type III porous liquid was reported in 2014, consisting of zeolitic imidazolate framework-8 (ZIF-8) suspended in glycol-2-methylimidazole solution, although it was classified as a slurry and, whilst it exhibited CO_2_ sorption, its permanent porosity was not reported.^39^ The first formally identified type III porous liquids can therefore be narrowed down to 2018, where three different systems were reported that all consisted of MOFs or zeolites dispersed in ionic liquids.^40–42^ First, Dai and co-workers reported the dispersion of ZIF-8, ZSM-5, and silicalite-1 in the ionic liquid [DBU-PEG][NTf_2_] to form stable type III porous liquids at room temperature (Fig. 2f),^40^ which was quickly followed by further examples of ZIF-8 dispersed in other alternative ionic liquids – commercially available [BPy][NTf_2_]^42^ and the phosphonium ionic liquid [P_6,6,6,14_][NTf_2_].^41^

The realisation of these initial type III porous liquids then opened the flood gates to a whole range of further examples being reported, ranging from similar ZIF-8 and ZSM-5 systems,^43–47^ to systems incorporating other MOFs and non-ionic size-excluded liquids. For example, in 2019 Li and co-workers reported that colloidally stable type III porous liquid dispersions could be formed in poly(dimethylsiloxane) (PDMS) by surface modifying UiO-66 particles using a controlled radical polymerisation technique,^48^ and in 2020 James and co-workers vastly expanded this family of porous liquids by screening a broad range of porous materials (including MOFs, zeolites, and a porous organic polymer) in a variety of non-ionic liquids (including oils and polyethylene glycols).^49^ While most of these systems relied on the use of a MOF, ZIF, or zeolite, as the porous material, more recently there have been examples of alternative pore carriers being used to form type III porous liquids, including the dispersion of HS nanospheres in dimethylaminoethoxyethanol,^50^ hollow carbon nanospheres in polymeric ionic liquids,^51^ and POC microparticles in a range of oils and ionic liquids.^52^

The utilisation of ionic liquids in the formation of porous liquids has several advantages, not least the negligible vapor pressure of most systems, and recently ionic liquids were also incorporated into type I and type II systems. In 2020, Nitschke and co-workers reported the first type I porous ionic liquid which incorporated a metal–organic cage (MOC) – by appending ionic liquid functionality to the periphery of the MOC, a neat liquid phase was obtained (Fig. 2g).^53^ In an alternative strategy to form porous ionic liquids, Dai and co-workers utilised a supramolecular complexation strategy to increase the solubility of POCs – by partially functionalising POCs with a carboxylate group, it was found that porous liquids could be formed on mixing the anionic cage with different crown-ethers as the solvent.^54^ Like the previously reported crown-ether functionalised POC that had increased solubility in 15-crown-5, this strategy also drastically increased the solubility compared to an unfunctionalised POC, offering an alternative approach to a type II porous liquid. In addition, it was reported that the anionic POC could be mixed with dicyclohexano-18-crown-6 to form a type I porous ionic liquid due to the observation of a single melting point – it should be noted however, that the anionic cage and the dicyclohexano-18-crown-6 were both solids prior to mixing, and a 1 : 3 ratio was used to form the liquid, suggesting that this system is likely a eutectic type II porous ionic liquid or even a porous solvate ionic liquid, *i.e.*, the cage salt and coordinating solvent are forming a complex with very similar properties to an ionic liquid.

Finally, most of the porous liquids introduced so far have been based on a subset of porous materials (*i.e.*, POCs, hollow silica/carbon spheres, MOFs, ZIFs, and zeolites), and focused on type I–III porous liquids. As the field continues to evolve, other porous solid materials have now started to become the focus of studies. For example, translation of a covalent organic framework (COF) into a type III porous liquid was realised by controlling the COF colloid size and functionalising the surface with tethered ionic liquids to form stable dispersions in room temperature ionic liquids (Fig. 2h).^55^ Another example involved the incorporation of an alternative discrete organic molecule containing a pore to form what is described as a type I porous liquid – cyclodextrins were post-synthetically modified with ionic liquid functionality to render them liquid (Fig. 2i).^56^ However, it is unclear if these cyclodextrin-derived liquids are true type I porous liquids or simply exhibiting favourable guest–host interactions – while no attempts have been made to directly quantify or investigate the porosity of this system, UV-Vis and circular dichroism spectroscopy suggested that self-filling from the pendant imidazolium does not occur. It was also only recently proposed that the initial types laid out in the original concept paper (types I–III) should be expanded to incorporate type IV systems^16^ – this was based on neat liquid MOFs being realised. In 2017, Coudert, Bennett and co-workers reported the structure of a liquid MOF, ZIF-4, which melted at 865 K, with computational modelling indicating that some of the porosity of the parent framework structure was maintained on melting to form a liquid (Fig. 2d).^57^ While this system was a liquid material with porosity, it did not initially fit neatly into the type I–III porous liquid types due to the transient nature of the pores and by its nature of being a melting framework rather than composed of discrete molecules or particles. Perhaps controversially, with this recently updated classification, these materials are now considered type IV porous liquids. However, one could argue that while the pores are transient, which goes against the original defined criteria for a material being a porous liquid over a conventional liquid, there is intrinsic porosity originating from the pore carrier, and the overall pore sizes are substantially larger than those observed in non-porous liquids. For example, in the liquid state, computational modelling suggested that ZIF-4 contains pore sizes of at least 2.4 Å,^57^ considerably larger than those in typical imidazolium ionic liquids which typically don't exhibit voids over 1.0 Å,^58,59^ and much larger than those in *n*-hexane (∼0.2 Å) and water (<0.7 Å).^60^

Through recent exploration of this new phase of microporous materials, many design considerations and techniques have been outlined, the largest being the importance of size-exclusion in selecting the components of each porous liquid. Not only has realisation of the four categories of porous liquids been achieved, in the case of type II and III porous liquids there are now a variety of different materials that could be selected for different potential uses, each accessed *via* differing synthetic approaches. This indicates that the field is approaching the end of its infancy. Going onward, the expectation to demonstrate the presence and extent of permanent microporosity in newly reported materials will become more important as these materials' compositions, as well as the techniques used to measure their properties, become more and more diverse. In addition, there will be an increasing need for key design principles to achieve both higher pore concentrations and therefore higher porosity, and larger pores that can host more or larger guest molecules. However, to realise porous liquids with higher pore concentrations or larger pores, a number of hurdles still need to be overcome. For example, while it has been possible to discover type II porous liquids with higher pore volumes, the resulting systems were highly viscous and started to undergo gelation at high concentrations.^27^ Type III porous liquids are perhaps the easiest systems with which to obtain higher pore volumes, although again, viscosity issues start to come into play at high concentrations especially when the material is closer to a slurry than a flowable dispersion. Technically, type I porous liquids offer the potential to access a material with the highest theoretical pore volume as a solvent would not be required for fluidity – however, the size of pendant groups used to induce melting needs to be considered, with higher molecular weight species leading to lower specific pore volumes, and likely high viscosity. The process of targeting porous liquids that maintain their microporosity but contain larger cavities presents additional challenges, for example, as the pores become larger more emphasis needs to be placed on the molecular design considerations that allow for solvent exclusion, *i.e.*, the solvents must be large enough, or the pore windows small enough, to prevent cavity occupation.

## 3. Characterisation of porous liquids

### 3.1 Experimental characterisation

While the field of porous solids is well established in relation to standard adopted methods for their characterisation and benchmarking their porosity, this is not necessarily the case for porous liquids. First, the techniques traditionally used for the analysis of porous solids are not always translatable to liquid state systems, especially type II and III porous liquids when they have an inherent vapor pressure. Second, it is not always common for the properties of the liquid phase to be thoroughly investigated, other than the porosity and gas uptake capability – for example, not all reports include solubility limits, viscosity, density, vapor pressure, or stability measurements, although this is all valuable information in order to properly assess porous liquids for different applications. In addition, for the porous liquids reported to date, a range of different methodologies have been used for their characterisation, meaning effective cross-comparison remains an ongoing challenge.

In the first examples of porous liquids, simply observing gas capacity improvements in the systems was not sufficient to verify the presence of permanent micropores – it was still unknown whether uptake enhancement occurred due to guest preference for the gas in the host cavity over other molecules in the liquid (*i.e.*, competitive binding), rather than the presence of permanent, accessible pores. Instead, other methods were used to confirm the presence, and where possible the nature, of the pores. Computational modelling and carefully selected control liquids, which contain similar functionality to the porous material but that lack a pore,^28,52^ have both been used to provide support for the presence of permanent porosity. However, positron annihilation lifetime spectroscopy (PALS) has emerged as an important technique for confirming the presence of permanent pores and their size in several examples of porous liquids. PALS relies on the generation of *ortho*-positronium within a sample and subsequent detection of the rate of decay of the positroniums as they pass through the material and can be applied to solid microporous materials.^61,62^ The introduction of unoccupied pores within the bulk of a material extends the lifetime of positroniums, and therefore increased lifetimes correlate with porosity. This approach was first applied to the crown-ether functionalised POC type II porous liquid, where the measured lifetime was an average of the lifetimes in the neat POC and neat 15-crown-5 solvent, suggesting the porosity was due to the contribution of both components – on fitting the data, the PALS measurements were consistent with the presence of permanent pores in the liquid.^26^ Since then, it has been successfully applied to other new classes of porous liquids to confirm the presence of permanent porosity, such as in zeolite^33^ and MOF^42^ particle-based type III porous liquids and in the type I MOC ionic porous liquid.^53^ However, it is worth noting that PALS experiments are typically carried out under high vacuum, meaning the technique is not suitable for porous liquid solutions or dispersions where the solvent has a significant vapor pressure.

While gas uptake measurements alone are not a definitive measure of permanent porosity in porous liquids, they are still of use in determining the available pore volume in a porous liquid. Conventional volumetric gas sorption analysis can be used on porous liquids with zero or near-zero vapor pressures, for example, the methane capacity in the crown-ether functionalised POC type II porous liquid was measured using a manometric gas equilibration method due to the low vapor pressure of the solvent used.^26^ Nitrogen isotherms were first reported in a porous liquid by Zhang *et al.* for their initial silica nanosphere porous liquid, allowing calculation of pore size distributions for this system.^27^ Subsequently, CO_2_ absorption isotherms have been measured in many porous liquids when the system consists of only components with little to no vapor pressure.^31,34,35,37,40,42,43,45,46,48,49,51,52^ Gravimetric absorption of CO_2_ and CH_4_ has also been measured in type III porous liquids,^41^ as well as water vapor sorption for surface modified surface modified UiO-66 based porous liquids.^48^ The latter, water vapor sorption, is an interesting method in that it can theoretically quantify the pore volume of certain porous liquid systems (*i.e.*, those that are stable to water) and also provide an indication of infiltration of the size-excluded solvent over time. It is also worth noting that an increase in CO_2_ uptake does not necessarily mean that permanent porosity is present in the liquid, for example, it is known that CO_2_ solubility in ionic liquids can vary based purely on the water content.^63,64^ However, many examples of type II porous liquids are prepared from relatively low-boiling solvents that would vaporise under vacuum conditions and contribute to a pressure increase within the instrument during gas sorption measurement.^28–30^ In these cases, exploitation of standard spectroscopic techniques can be used to observe gas capacity, for example, infrared and nuclear magnetic resonance spectroscopy have been used to determine relative and quantitative gas uptakes respectively in porous liquids.^28–30^ Beyond this, having an understanding of the occupation preference of liquid guests over gaseous guests in porous liquids can be used to chemically displace the gas occupying the pores, the volume of which can be captured and measured to determine the quantity of gas in the porous liquid. This gas release technique ultimately has limitations compared to directly measuring the volume of gas absorbed, as the volume measured is indirectly linked to the total capacity of the material.^28^

**Fig. 3:**
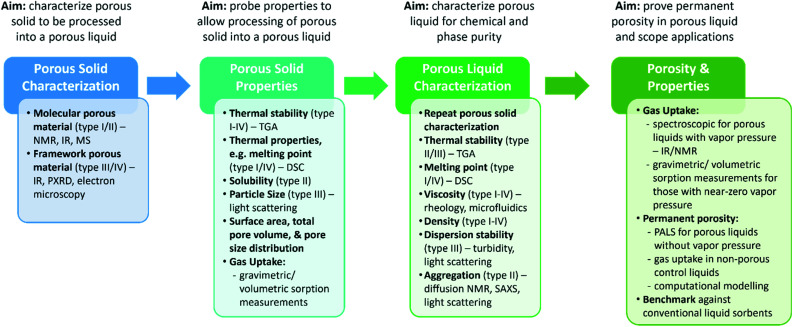
Overview of the porous liquid discovery process, and the recommended characterisation required at each stage.

Overall, with the majority of porous liquids so far being based on either microporous molecular or framework materials, standard characterisation techniques for the solids are typically employed – many researchers have a background in a specific type of porous solid material which they then attempt to develop into porous liquids. On preparation of the porous liquid, similar analysis is then often carried out to determine how the porosity is changed on translating the material into a liquid or flowable state, although this is not always suitable. In addition, the properties of the liquid itself, including the viscosity, density, thermal properties, and stability, which are arguably just as equally important to the gas uptake, are often not studied for new porous liquids. We therefore propose that at each stage of the porous liquid discovery process, a minimum set of characterisation is required (Fig. 3). First, this will ensure that the porous solid that is to be processed into a porous liquid is fully characterised in terms of its composition, structure, and purity. Second, the relevant properties for the type of porous liquid that the porous solid will be incorporated into need to be studied – for example, the thermal stability of the porous solid is important across all types, whereas the thermal properties are more important for type I and IV systems, the solubility is important for type II porous liquids, and the particle size for type III porous liquids. In addition, the porosity and gas uptake of the porous solid should be investigated, ideally for the same gases of interest in the porous liquid, as this can have a direct impact on the overall porosity of the resulting porous liquid. For example, this is especially relevant for type III porous liquids where the porous solid will be dispersed in a liquid. This ensures that there is a good understanding of the porous solid itself and how the properties might transfer into the liquid state. If the total pore volume and pore size distribution of the solid porous material is also obtained and reported, for example using nitrogen adsorption, then calculating the estimated pore volume of the subsequent porous liquid can be relatively simple. Once a porous liquid has been formed, further characterisation should then be carried out to both confirm the chemical and phase purity, and to understand the physical properties of the liquid itself – for example, the thermal properties (including the thermal stability and melting point), viscosity, and density are important across all types, whereas the physical stability in relation to aggregation or creaming/sedimentation are important for type II and type III systems respectively. Finally, once the liquid itself has been characterised, the gas uptake should be investigated, and the permanent porosity of the liquid should be confirmed to ensure a porous liquid has been formed. At this stage, it is also important to compare the uptake and separation capabilities of the porous liquid against its constituent components – this shows which properties are transferred to the liquid state, and which are not, and is very important for understanding the nature of porosity in these materials. We envisage that this step-by-step experimental approach will hopefully mean that drawing conclusions and cross-comparison of porous liquid materials becomes easier in the future.

### 3.2 Computational characterisation

**Fig. 4:**
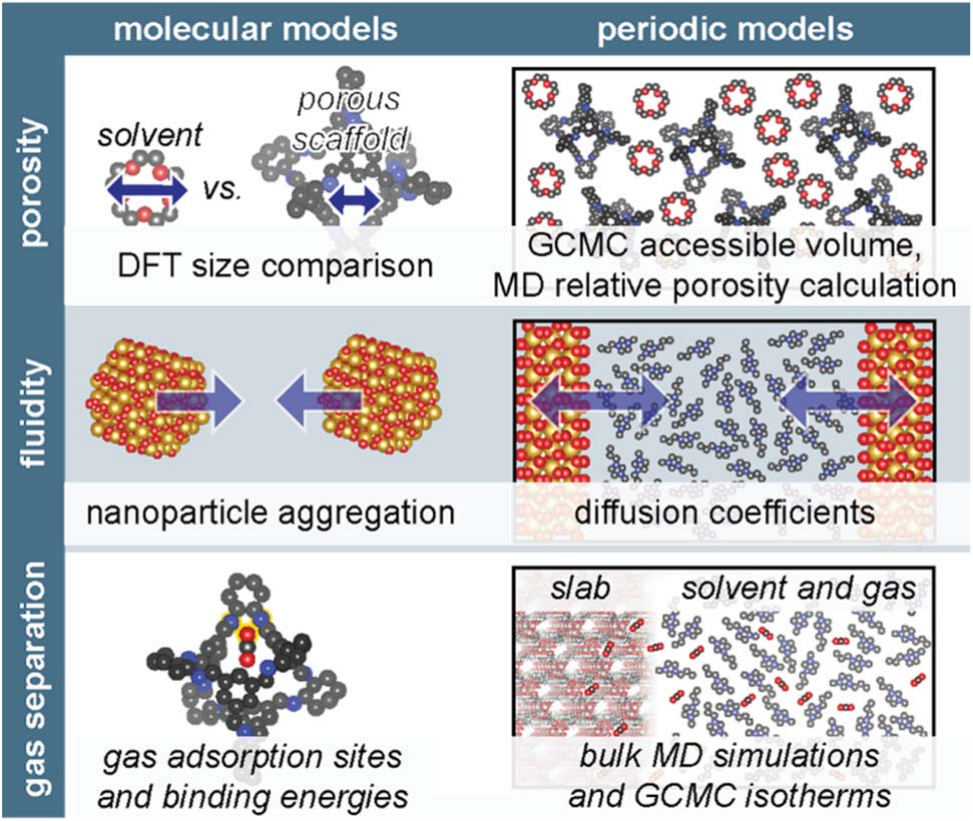
Common molecular (left panel) and periodic (right panel) models and methods are briefly outlined for three porous liquid properties; porosity (top), fluidity (middle), and gas adsorption (bottom). Hydrogens omitted for clarity.

Often, experimental efforts are complemented by computational studies, which offer atomistic insight into observed phenomena. Yet, measurable properties of porous liquids arise from interactions and phenomenon occurring at varying time and length scales,^65^ necessitating models from classical to quantum chemical calculations. The diverse composition of porous liquids affords a series of model types; common porous liquid models and studied properties are outlined in Fig. 4. For example, within a single study, hollow silica-based type I porous liquids were modelled as discrete nanoparticles to examine aggregation, as well as slabs to ascertain qualitative diffusion properties.^66^ Type III porous liquids featuring homogeneously dispersed framework materials or nanoparticles of sufficient diameter (>20 nm) employ slab models because they allow increased resolution of surface-based phenomena, which is instructive for examining the interface between solvents and the extended framework solid in these porous liquids,^45^ including potential solvent ordering at the porous scaffold surface.^41^ However, the molecular nature of POCs in type II porous liquids does not allow for the periodic description by slab models; instead, studies either model POC-based porous liquids *via* bulk simulations,^26^ or use single cage models to ascertain individual interactions between solvent/gas molecules and the cage.^67^ Model selection is a function of both the chemical composition (*i.e.* extended framework *vs.* molecular material) and the feature(s) of interest.^68^ Within the porous liquid field, computational efforts are largely focused on describing several key properties: (i) porosity; (ii) fluidity; and (iii) gas uptake and separation capabilities.

#### Relative porosity

The permanent, intrinsic porosity of porous liquids is a critical characteristic differentiating them from conventional liquids. Thus, determining the origin of measured porosity is critical; yet as discussed, this is often experimentally challenging. Since pore availability is dictated by the relative sizes of the porous component and solvent molecules, rudimentary porosity analyses rely on size comparisons. Indeed, Dai and co-workers compare sizes of density functional theory (DFT)-optimised solvent molecules with pore channel widths to validate solvent selection in type III porous liquids.^40^ More commonly, molecular dynamics (MD) and grand canonical Monte Carlo (GCMC) simulations are employed. At the simplest level, bulk MD simulations featuring experimental porous liquid concentrations may be used to visualise the diffusion of solvent and offer an initial porosity prescription.^48^ Beyond visualisation, the percentage of solvent molecules located at pore windows and distribution densities is indicative of both the solubility and size-exclusivity.^28,69^ Perhaps more robust, radial distribution functions (RDFs) for a type I porous ionic liquid revealed solvent molecule proximity to POC windows; cavity size distributions were used to further validate RDF results.^54^ Number density profiles have also been used to show that gases preferentially occupy the cavities in a type III porous ionic liquid.^44^ GCMC simulations provide fractional accessible volumes using gas probe molecules and the gas adsorption density field distributions.^35,69^ Fractional free volume may be assessed *via* calculated insertion probabilities from MD trajectories,^26^ and has been paired with free energy calculations to determine the energetic cost of introducing a single solvent molecule^26^ or the terminal chain of a neighboring POC^24^ into a POC cavity using umbrella sampling with the weighted histogram analysis method.

#### Suspension stability, dispersion, and fluidity

In addition to offering permanent porosity, porous liquids maintain the fluidity and mass transfer capabilities of liquids. Liquid-like disorder, and hence fluidity, may be probed using partial RDFs from MD trajectories, as demonstrated for type IV porous liquids composed of liquid ZIF-8;^57^ here, liquid-like disorder was concluded because the RDF is non-zero after the first peak, and viscosity, an important measure of fluidity, was estimated from translational diffusion coefficients of the Zn cations and imidazolate anions of ZIF-8 from first principles MD simulations.^57^ Diffusion coefficients may also be obtained from mean squared displacement over time.^26^ The aggregation propensity is one indicator of porous liquid stability; recently, this was assessed by tracking the distance between hollow SiO_2_ nanoparticles in water during an MD simulation.^66^ The effect of functionalising the SiO_2_ nanoparticles with varying polymer chains on mobility, aggregation, and dispersion, was subsequently analysed using a model containing two slabs separated by solvent molecules. By tracking the distance between the slabs during equilibration, a qualitative understanding of fluidity was achieved.^66^ The closer the slabs were, the less fluid the porous liquid. The authors later included the pores of the porous liquids and examined the depth to which canopies of the grafted SiO_2_ nanoparticles entered the pores.^70,71^ Assessing suspension stability is specifically necessary when forming type III porous liquids from porous solids; solvent molecules must not only be excluded from the pores, they must stabilise the porous scaffolds.^41^ Indeed, charge transfer from the alkyl chains of [P_6,6,6,14_][Br] to the zeolite H-ZSM-5 upon alkyl chain penetration helps balance van der Waals forces and mitigates aggregation of the porous liquid in chloroform.^45^

#### Selective gas uptake

Gas storage and separation are among the leading applications for porous liquids; computations ranging in scale are used to assess porous liquid performance by confirming gas preferentially occupies cavities,^44^ supporting solubility measurements,^26^ and identifying specific adsorption sites,^35^ among other utilities.^72^ MD simulations demonstrate the impact of gas separation *via* RDFs, and spatial distribution functions have revealed energetics and structure are the driving forces behind transferring gas from the ionic liquid to the MOF in a type III porous liquid.^44^ Spatial distribution functions have also been used to probe the topology–geometry relationship between gas and POCs within a type II porous liquid, revealing which gases are preferentially bound to the cage cavity.^72^ In a similar approach, the isosurface of gas occupancy within the cavities of POC-based porous liquids were calculated from MD trajectories to examine gas entrance kinetics.^73^ The energy barrier of gas entrance/exit from cage cavities was calculated using potential of mean force (PMF) of gas molecules near and inside the cavities; the existence of an energy penalty for leaving the cage suggests the cage can trap gas. This was corroborated *via* the occupancy autocorrelation function of gas molecules, which identifies the time scale that a gas molecule is trapped within the cavity.^73^

The energetic emphasis of GCMC calculations is advantageous for gas adsorption calculations of porous materials.^74^ GCMC have been used to examine CH_4_ solubility in a POC-based type II porous liquid,^26^ as well as identify CO_2_ selective sites in a MOF-based type III porous liquid,^35^ and a hollow silica type I porous liquid.^75^ GCMC isotherms have also been compared with experiment to verify that PDMS chains within the MIL-101(Cr) cavity disrupted the gas uptake process by calculating the isotherm of a model featuring PDMS manually placed within the MOF pores.^48^

The detailed information offered by DFT provides additional insight into the mechanism of gas uptake. Yet, the increased computational expense of DFT necessitates smaller models, typically composed of two to three porous liquid components. For example, binding energies are calculated to examine specific molecular interactions, including those between the gas/porous motif,^67,76^ gas/solvent and gas/porous liquid,^77,78^ and solvent/porous motif.^67,79^ Beyond binding energies, exchange energies of CO_2_/CHCl_3_ in a series of POCs provided a detailed picture of CO_2_ separation by type II porous liquids.^67^ These studies take further advantage of increased electronic resolution to visualise weak interactions using the reduced density gradient analysis,^67,80,81^ quantum theory of atoms-in-molecules,^82^ and independent gradient model.^56^ Beyond these procedures, electrostatic potential maps offer critical information regarding the electron density of porous liquid components^56^ and potential adsorbates;^67^ these methods have recently been used as a screening metric for ionic liquids – an important component of many type III porous liquids.^83^

Overall, the chemical diversity of the porous liquid field affords an array of computational model configurations, which are ultimately dictated by the porous liquid type and feature(s) of interest. The discrete, molecular components of type I and II porous liquids motivate models that take advantage of their simplicity – often featuring single-molecule cluster models – to obtain a detailed description. Alternatively, simulations featuring bulk solutions at varying concentrations may also be achieved. Type I porous liquids composed from hollow nanospheres may also benefit from slab models that provide atomistic insight into the interface between the nanosphere surfaces and solvents. Slab models are also advantageous when modeling type III porous liquids, allowing adequate assessment of the interaction between the framework dispersions and size-excluded liquids. Perhaps the most challenging systems to model are type IV porous liquids. In addition to being the newest recognised type, their amorphous nature requires larger models, and subsequently, more computational resources to adequately describe the long-range disorder within the system. As such, simpler cluster models are not appropriate in this instance. Ultimately, the breadth of methodologies and models employed to date are limited by the youth of the porous liquid field. Indeed, as additional functionalities are discovered and additional applications are envisaged, computational tools will expand as well.

## 4. Applications of porous liquids

**Fig. 5:**
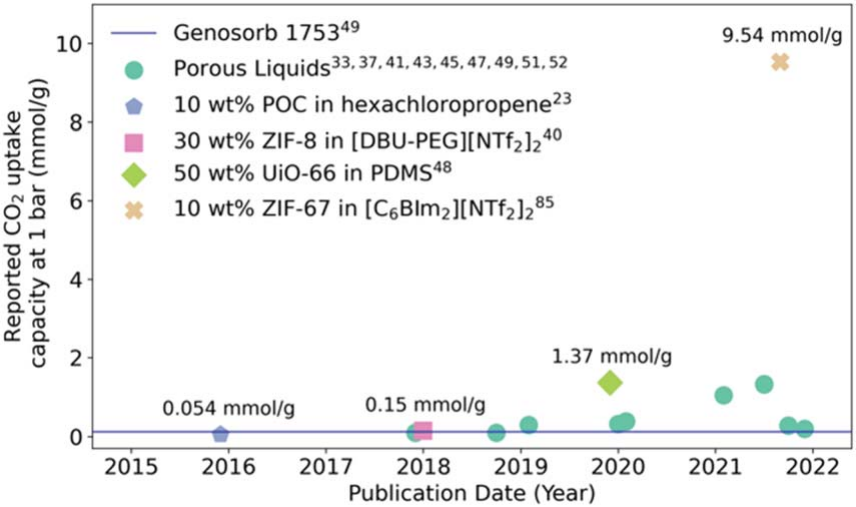
Reported CO_2_ uptake capacities in porous liquids at *ca.* 1 bar and *ca.* 298 K over time. A selection of the significant developments are highlighted to show the acceleration in progress.

The most reported potential application of porous liquids is in gas uptake and selective gas separations. Due to the infancy of the field, in addition to different design strategies being reported to form porous liquids, a major challenge has been the formation of porous liquids with higher pore volumes, and therefore higher gas uptakes. For example, based on the potential for porous liquids to be an alternative liquid absorbent for carbon capture,^84^ the uptake of CO_2_ has been reported for a large number of these systems (Fig. 5). CO_2_ absorption capacity reflects the capability these materials have to compete with other industrial gas sorbents, for example comparisons have been made between porous liquids and Genosorb 1753 due to the latter's capacity for absorbing CO_2_ in the natural gas sweetening process.^20^ The best performing systems demonstrated so far are type III porous liquids based on MOF particles with significant CO_2_ adsorption capacity – some of the most effective are prepared from ZIF-8,^40^ ZIF-67,^85^ Al(fum)OH^49^ and UiO-66.^48^ Overall, since their first realisation in 2015, the CO_2_ uptake capacity in porous liquids has drastically improved compared to the earliest examples (Fig. 5). While one of the first POC based porous liquids exhibited improved CO_2_ uptake, the high molecular weight means that the overall pore concentration is relatively low considering the mass of cage present (10 wt%), so CO_2_ uptake capacity is limited.^26^ Framework materials used in type III porous liquids are generally much more effective for generating high CO_2_ uptakes, and since porous liquids were first reported, these have had the greatest CO_2_ capacity of all reported porous liquids to date. When compared to Genosorb 1753, porous liquids quickly surpassed the CO_2_ capacity at 1 bar, although it should be noted that generally the process in which Genosorb 1753 is used occurs at much higher working pressures. The highest CO_2_ uptake seen so far is in a porous liquid prepared from ZIF-67 particles dispersed in a bulky ionic liquid – this exceptionally high reported uptake enhancement over the neat ionic liquid (>9 mmol g^−1^) does not reflect observations reported elsewhere in the literature, where the ionic liquid will usually make a significant contribution to the CO_2_ absorption capacity, while also not reflecting the reported CO_2_ capacity of solid ZIF-67 (0.61 mmol g^−1^),^86^ indicating some hybrid synergistic mechanism may be occurring.

In addition to the selective capture of CO_2_, porous liquids have also recently been exploited for CCS and carbon capture and utilisation (CCU).^87,88^ A recent work demonstrated how the adaptation of HS nanorod porous liquids can be combined with carbonic anhydrase, a metalloenzyme effective for converting CO_2_ to CaCO_3_, as a material for CCS.^87^ The enzyme was suspended in the OS portion of the porous liquid, resulting in a fluid material with high CO_2_ uptake and the ability to sequester CO_2_. A method for enhancing CCU through use of porous liquids has also been recently reported, where addition of ZIF-8 to a catalytic ionic liquid not only increased the CO_2_ uptake, it greatly increased its catalysis with epoxides to form cyclic carbonates, as well as increasing the overall yield.^88^

The separation and selective uptake of other gaseous molecules has also been studied in porous liquids, for example, modification of the pore size and shape in POCs in a type II porous liquid makes it possible to control gas uptake and selectivity for small gases – Xe uptake enhancement was effectively eliminated in a porous liquid using this method.^30^ Similarly, James and coworkers recently demonstrated how combining selected porous components and carrier liquids in type III porous liquids is effective in tuning gas uptake selectivity.^89^ In type III porous liquids, since consideration of solubility does not need to be made, almost any fluid phase can be selected. In this case, in order to maximise C_2_H_4_/C_2_H_6_ selectivity, Ag containing materials were selected for both the porous solid and fluid phase,^89^ and while the selectivity for C_2_H_4_ over C_2_H_6_ at 0.8 bar was reduced in the porous liquid (25.6) compared to the neat liquid (33.13), the overall capacity increased almost 4-fold at this pressure. In addition, Nitschke and co-workers' neat MOC porous ionic liquid was found to encapsulate chlorofluorocarbons (CFCs) from the gas phase,^53^ as observed previously in analogous solid cages.^90^

More recently, the ability of porous liquids to be used in molecular separations of non-gaseous molecules has begun receiving interest. Previously, enantioselectivity in a type II porous liquid was investigated based on the fact that analogous solid POCs to those used in the porous liquid had been shown to be effective;^91^ despite this, no enantiomeric excess was observed when carrying out the same method as with the solid POC.^28^ This reflects the fact that the mechanism of this separation was due to features of the extended pore network in the solid material, which are not present in a type II porous liquid. However, chiral resolution in a porous liquid has since been achieved – Wang and co-workers' cyclodextrin based type I porous ionic liquid was successfully applied in the enantio-resolution of racemic nucleosides.^56^ In particular the researchers showed that the γ-cyclodextrin example could separate the enantiomers of β-2′-deoxycytidine to an enantiomeric excess of 84.8%. Similarly, with Nitschke and co-workers' neat MOC porous ionic liquid, the internal structure of the pore exhibited strong size and shape selectivity for aliphatic C_3_- and C_4_-alcohols with different extents of branching – the cavity was more selective towards branched alcohols, with *tert*-butanol most preferable as its shape and size better satisfy the cavity volume.^53^ Molecular selectivity has not only been achieved in molecular porous liquids; 2,2,3,3-tetrafluoro-1-propanol (TFP) can be removed from a 3 : 1 mixture of TFP and water by liquid–liquid extraction with a type III porous liquid prepared from ZIF-8 dispersed in the ionic liquid [BMIm][NTf_2_].^92^ The extraction efficiency from the mixture was found to be generally comparable with the neat ionic liquid in some cases, but the total extraction capacity is improved with the introduction of porous media, as well as improving the extraction efficiency at lower loadings of TFP in the extractant mixture.

As well as being useful for separations, porous liquids have shown potential for absorption of volatile organic compounds such as toluene, owing to the presence of the permanent pores allowing for significant uptake of these small molecules.^85^ A significant step towards utilisation of porous liquids in industry sees the use of ZIF-8 dispersed in [BPy][NTf_2_] as a medium for extracting thiophene and benzothiophene from a model oil mixture.^93^ Using this material, the desulfurisation efficiency was greatly improved relative to the neat ionic liquid, and the researchers also demonstrate that the improvement is due to the presence of permanent porosity in the ZIF-8, as when an ionic liquid with a less bulky anion is used much of this enhancement is lost.

Beyond exploiting the molecular selectivity of microporous materials for separations, introduction of catalytic components into porous liquids has been explored recently. The design of porous liquids with catalytic components can combine the benefits of porous heterogeneous catalysts for size and shape selective sorption with increased gas solubility in the liquid phase, compared to non-porous or non-liquid catalysts, to create more selective or efficient catalysts. The encapsulation of metal (Au, Pt and Pd) in silica nanosphere based type I porous liquids followed by surface functionalisation with OS/PEGS corona has been reported as a technique to form fluidised metal heterogeneous catalysts by Maschmeyer and co-workers.^94^ The authors then demonstrated hydrogenation of various substrates using the Pt-encapsulated porous liquid catalyst, finding that these materials behave similarly to other encapsulated metal nanoparticles,^95^*i.e.*, the reaction is kinetically limited by the encapsulant and surface corona limiting mass transfer to the metal surface.^96^ No driving force due to permanent porosity was observed in the porous liquids, but when carried out in ethanol, the reaction proceeded more quickly due to the lower viscosity of this solvent aiding diffusion of the substrate. The researchers also show that techniques effective with bulk silica can be used to immobilise Pd N-heterocyclic carbene complexes on the internal surface of these silica nanosphere porous liquids, and this was then used as the catalyst in a Heck reaction between iodobenzene and butyl acrylate.^97^ More recently, Dai and co-workers reported the use of a bifunctional type III porous liquid containing both acidic and basic sites which successfully catalysed a cascade deacetalisation–Knoevenagel/Aldol condensation in one pot.^98^

Many of the properties of the components found in porous liquids overlap with the desirable properties of materials useful for porous membranes, for example the solution processability of POCs makes them ideal candidates for processing into supported^99^ and mixed matrix membranes (MMMs).^100^ Similarly, the ability to prepare colloidal MOF particles to form suspensions enables controlled deposition from suspensions in a similar manner.^4^ Supported liquid membranes make up another category of membranes with potential use in gas separations.^101^ In the work of Deng *et al.*, the MOC based type II porous liquid was prepared as a liquid membrane with graphene oxide as a support.^69^ Through measurement of the permeance of CO_2_, H_2_ and N_2_ in this membrane compared to the neat 15-crown-5 solvent, it was found that the presence of permanent pores in the membrane dramatically enhanced diffusion through the membrane, more significantly improving H_2_ permeance (4.0 times) over CO_2_ (3.0 times) and N_2_ (2.7 times). Porous liquids can also be included as a component in a MMM, for example, a HS nanosphere based porous liquid was incorporated into a polymer matrix at up to 25 wt% in order to enhance the CO_2_ permeability and CO_2_/N_2_ selectivity.^75^ The use of the porous liquid was required in this case, as unmodified silica spheres agglomerated on preparation as a membrane.

## 5. Conclusions

The field of porous liquids has seen rapid growth in the last few years, with new examples of the different types and new applications being reported more frequently, and more porous, porous liquids being experimentally realised – this is only likely to grow further as the field progresses. One particularly exciting prospect is the utilisation of porous liquids in place of conventional liquid sorbents, although this is likely to focus on type II and type III porous liquids due to the inherent high viscosities of neat type I and IV systems which may be problematic in flow applications. In addition, while porous liquids can currently exceed the capacity for carbon dioxide compared to industrially used liquid sorbents at low temperatures and pressures, several properties such as their stability, viscosity, selectivity, recyclability, cost, and scalability, still need to be optimised in order for such systems to be competitive replacements. Part of this will come down to solvent selection and design, especially as for some applications it is preferable for the porous material and liquid components to have complementary properties, for example in James and coworkers type III porous liquid, a silver-containing zeolite and silver-containing ionic liquid were selected to maximise selectivity for C_2_H_4_ while also maximising capacity.^89^

As the field progresses, it can also be envisioned that a combination of high-throughput experimentation and computation will position porous liquid design and optimisation at the forefront of separation materials research, although the characterisation of porous liquids using standardised methodologies so new systems can be benchmarked against a well-characterised library is currently lacking. It is also worth considering whether computational modelling alone provides sufficient evidence to verify the permanent porosity and gas uptake capability of a porous liquid, or whether this should be demonstrable experimentally. Considering the properties of the liquid, rather than mainly focusing on the gas uptake capabilities, will also provide us with a much deeper understanding of the structure–property relationships in porous liquids and determine their suitability for different applications. For example, viscosity will not only play a role in the ability to pump porous liquids, it may affect gas diffusivity and therefore the overall uptake and retention of different gases; density measurements should give an indication of the porosity of a liquid with porous liquids with higher pore volumes being less dense; vapor pressures may limit operational working temperatures and pressures; and an understanding of the stability of the different porous liquids, whether thermal (type I and IV), precipitation *vs.* gelation (type II), or creaming *vs.* sedimentation (type III), will be important for subsequent applications.

## Author contributions

The manuscript was written through contributions of all authors. All authors have given approval to the final version of the manuscript.

## Conflicts of interest

There are no conflicts to declare.

## Supplementary Material
